# Epidemiology of Firework-Related Eye Injuries From 2002 to 2021

**DOI:** 10.7759/cureus.104122

**Published:** 2026-02-23

**Authors:** Jay Jaber, Molly Pluenneke, Austin Huang, Madison Zhao, Andrew G Lee

**Affiliations:** 1 Department of Ophthalmology, Baylor College of Medicine, Houston, USA; 2 Department of Research, Rice University, Houston, USA; 3 Blanton Eye Institute, Houston Methodist Hospital, Houston, USA

**Keywords:** emergency ophthalmology, eye injury prevention, firework injury, ocular chemical burns, ocular trauma, pediatric eye trauma, protective eye gear, visual impairment

## Abstract

Background

Improper use of fireworks can lead to traumatic eye injuries, especially around the Fourth of July. This study investigates emergency department presentations of firework-related eye injuries across demographic factors and identifies notable injury trends over the last 20 years using the National Electronic Injury Surveillance System (NEISS).

Methodology

We queried the NEISS database for firework-related eye injuries from 2002 to 2021 using product code 1313 and body part codes 77 (eye) and 76 (face). Cases were stratified by age, sex, month, year of injury, and injury type for descriptive statistical analysis. Spearman’s rank correlation was conducted to assess annual trends.

Results

Between January 1, 2002, and December 31, 2021, there were 1,213 reported cases in NEISS, corresponding to an estimated 41,708 (95% confidence interval = 31,913-51,500) emergency department visits nationally. Of these, 871 (71.8%) occurred in males and 342 (28.2%) in females. Patients aged ≤18 years comprised 752 (62.0%) cases, including 327 (27.0%) children aged 6-11 years. Contusions were the most common injury type (484 cases, 39.9%). The majority of injuries occurred in July (850 cases, 70.1%). Annual injury totals demonstrated a weak negative, statistically non-significant correlation over time (r = -0.26, p = 0.268), with notable increases in 2005, 2020, and 2021.

Conclusions

When both eye and facial injury codes are included, periocular contusions represent the most common firework-related ocular trauma, differing from prior studies that reported thermal burns. The disproportionate burden of injury among minors and the strong seasonal clustering in July underscore the need for enhanced preventive education and regulatory measures surrounding consumer fireworks.

## Introduction

Around the world, fireworks are used to mark momentous occasions, and specifically in the United States, to celebrate the Fourth of July. Many Americans attend firework display shows or purchase consumer home fireworks to commemorate Independence Day.

The use of fireworks is often associated with injury, with some studies reporting that 31% of firework-related trauma affects the eye [1]. To monitor the safety of fireworks and other consumer products, the National Electronic Injury Surveillance System (NEISS) established a database comprising a nationally representative sample of hospital-reported injuries (Publicly Available Database: U.S. Consumer Product Safety Commission. National Electronic Injury Surveillance System (NEISS) Injury Data; accessed February 5, 2023). The database is accessible for investigators to query datasets on desired demographics and perform statistical analyses.

The NEISS database is a public database offered by the United States Consumer Product Safety Commission. The database compiles injury reports from over 100 participating hospitals, using trained physician coordinators to document and standardize emergency department visits related to consumer products with the primary purpose of collecting detailed reports regarding these visits. The database is used as an epidemiological tool that allows individuals to access and stratify data based on various demographics, including treatment dates, product codes, age, sex, body part, diagnosis, and disposition (Publicly Available Database: U.S. Consumer Product Safety Commission, 2023) [2].

By analyzing the prevalence of firework-related eye injuries that have been reported to emergency departments from 2002 to 2021, this study aims to discover underlying relationships between these injuries and age, sex, month of injury, year of injury, and injury type. Additionally, the study seeks to uncover year-to-year trends of firework-related eye injuries and determine if the COVID-19 pandemic had a significant impact on the prevalence of reported firework-related eye injuries. Furthermore, given that periocular injuries may be underrepresented when relying solely on primary eye injury codes, this study aims to compare injury patterns using comprehensive case ascertainment methods, including both eye and facial injury codes supplemented by narrative review, to more fully capture the spectrum of firework-related ocular and periocular trauma.

## Materials and methods

Study design

This study is a retrospective epidemiologic analysis of firework-related eye injuries using the NEISS, a publicly available, nationally representative database maintained by the U.S. Consumer Product Safety Commission.

Data source

NEISS collects injury data from approximately 100 participating hospitals selected as a stratified probability sample of U.S. hospitals with 24-hour emergency departments and at least six inpatient beds. Each case is assigned a statistical weight, allowing national injury estimates to be calculated.

Study population and case identification

Firework-related injuries were identified using product code 1313. Cases from January 1, 2002, through December 31, 2021, were included. To capture ocular and periocular trauma comprehensively, we included body part code 77 (eye globe) and body part code 76 (face). Narrative descriptions were manually reviewed to confirm ocular or orbital involvement. Cases were included if the narrative referenced injury to the eye, eyelid, orbit, periorbital region, or vision, and excluded if injuries were limited to non-ocular facial structures.

Outcome measures

The primary outcome is a national estimate of firework-related eye injuries, and secondary outcomes include demographic distribution, seasonal variation, injury type distribution, and temporal trend analysis.

Statistical analysis

A 20-year dataset was created with four sets of five-year intervals: 2002-2006, 2007-2011, 2012-2016, and 2017-2021. Analyses were conducted using Microsoft Excel and R Studio (Version 2022.07.02). All analyses incorporated NEISS-provided sample weights to generate national estimates. Continuous variables were summarized using means and medians, and categorical variables were reported as frequencies and percentages. Temporal trends in annual injury frequency were evaluated using Spearman’s rank correlation. 

Ethics statement

This study utilized publicly available, de-identified data and did not involve human subjects as defined by federal regulations. Hence, Institutional Review Board approval was not required.

## Results

The NEISS query identified 1,662 firework-related injuries involving the face or eye between 2002 and 2021. Of these, 1,003 cases were coded under body part code 77 (eye globe), 627 under body part code 76 (face), and 32 under other body part codes. All narrative descriptions were manually reviewed to determine ocular or orbital involvement. Following a qualitative review, 197 of the 627 facial injuries and 13 of the 32 injuries coded to other body parts were determined to involve the globe or periocular structures. All 1,003 cases coded under body part code 77 were retained. This resulted in a final analytic cohort of 1,213 firework-related ocular injuries.

Notably, 210 of the 1,213 (17.3%) cases were not originally coded under body part code 77 but were identified through narrative review of facial and other injury codes, underscoring the importance of comprehensive case ascertainment when evaluating ocular trauma using NEISS data.

The 1,213 cases of firework-related eye injuries reported by NEISS participating hospitals from 2002 to 2021 can be extrapolated to an estimated 41,708 (95% confidence interval (CI) = 31,913-51,500) emergency department visits during this time frame across the United States. Injuries were relatively evenly distributed across the five-year intervals, each accounting for approximately one-quarter of total estimated cases (Table 1), with the highest percentage attributed to the years 2002-2006 at 27.4%.

**Table 1:** Total estimated eye injuries related to fireworks in the five-year intervals in which data were collected, along with the total value of nationally estimated injuries for the 20-year span from 2002 to 2021.

5-year interval	National estimates	Percentage of injuries by 5-year timeframe
2002–2006	11,441	27.40%
2007–2011	9495	22.80%
2012–2016	9462	22.70%
2017–2021	11,311	27.10%
Total	41,708	100.00%

**Figure 1:**
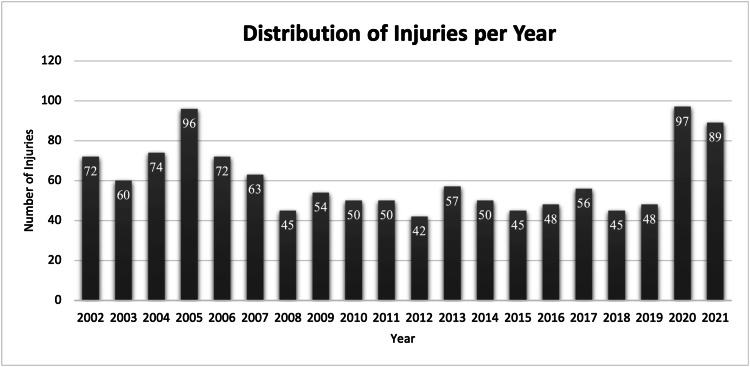
Reported National Electronic Injury Surveillance System (NEISS) emergency department visits from 2002 to 2021 for firework-related eye injuries.

**Figure 2:**
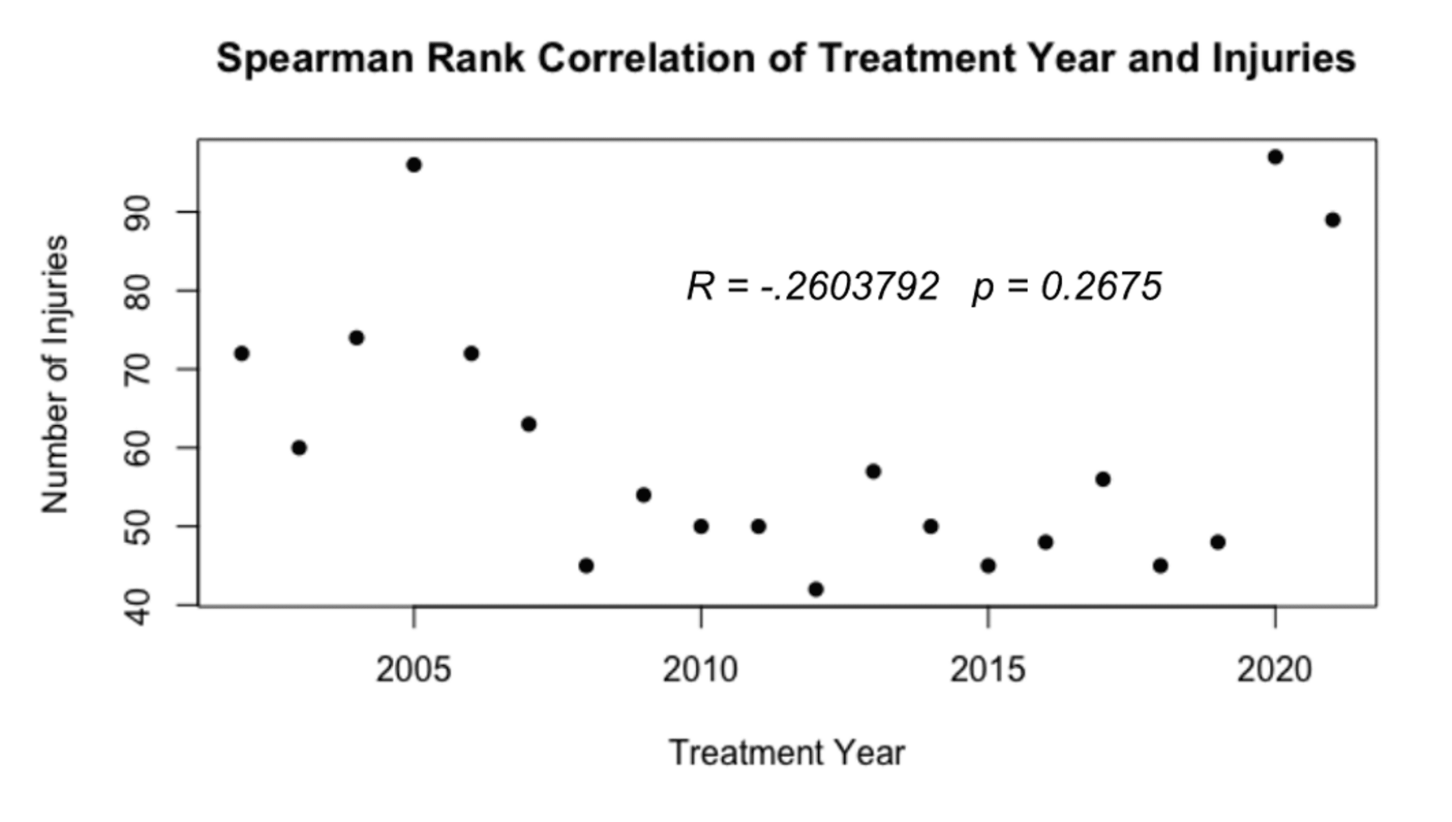
Spearman’s rank correlation between treatment year and reported injuries.

The data were further broken down into single-year periods. Annual national estimates ranged from a low of 42 cases in 2012 to a high of 97 cases in 2020, with a median of 55 cases per year (Figure 1). Spearman’s rank correlation analysis demonstrated a weak, non-significant negative association between treatment year and injury frequency (r = −0.26, p = 0.268) (Figure 2).

**Figure 3:**
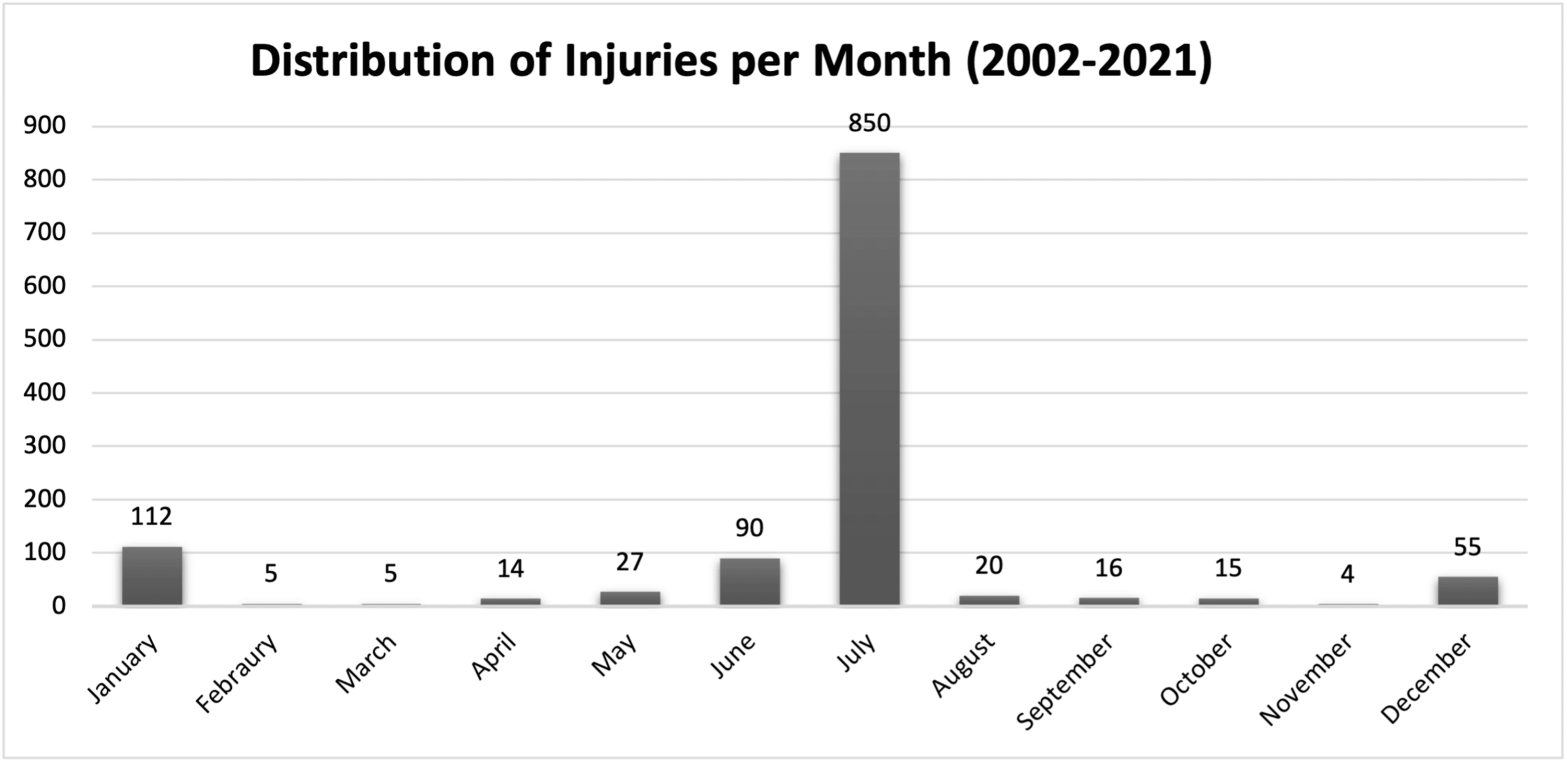
Bar chart plotting the distribution of reported firework-related eye injuries by treatment month from 2002 to 2021.

The 1,213 cases of firework-related eye injuries were also stratified based on the month the injury occurred. Over the last 20 years, 850 (70.1%) cases of the reported injuries occurred in July. The second and third most common months were January and June, with 112 (9.2%) and 90 (7.4%) injuries, respectively (Figure 3).

**Figure 4:**
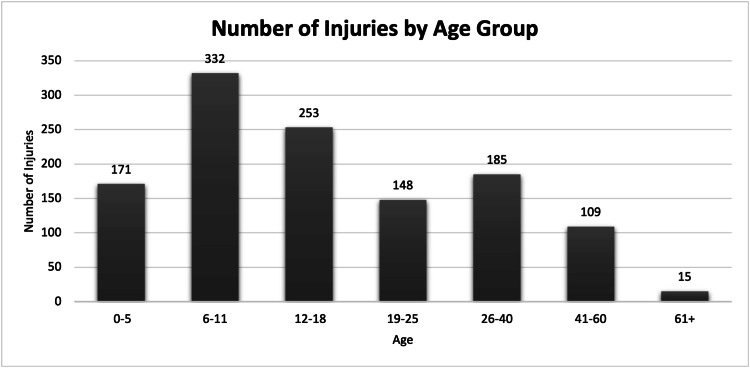
Bar chart plotting the distribution of reported firework-related eye injuries by treatment age group for the years 2002-2021.

Patients’ ages were stratified into seven groups based on ranges used in previous studies: young children (0-5 years), children (6-11 years), adolescents (12-18 years), young adults (19-25 years), adults (26-40 years), middle-aged adults (41-60 years), and elderly adults (≥61 years) [2]. Patients aged ≤18 years represented the largest age group, accounting for 756 (62.3%) injuries. Within this population, children comprised the greatest proportion of injuries (332, 27.4%), followed by adolescents (253, 20.9%), and adults (185, 15.3%). Elderly adults accounted for 15 (1.2%) cases (Figure 4).

From the 1,213 reported ED visits, 871 (71.8%) patients were of the male sex. The median age of firework-related injuries in males was 14 years old, and the mean age was 23.72 years. The remaining 342 (28.2%) patients were of the female sex and had a median age of 15 years and a mean age of 20.81 years (Table 2).

**Table 2:** Number of injuries and ages based on the reported sex of the patient.

Sex	Number of injuries	Percentage of injuries	Median age	Mean age
Male	871	72%	14	23.72
Female	342	28%	15	20.81

**Figure 5:**
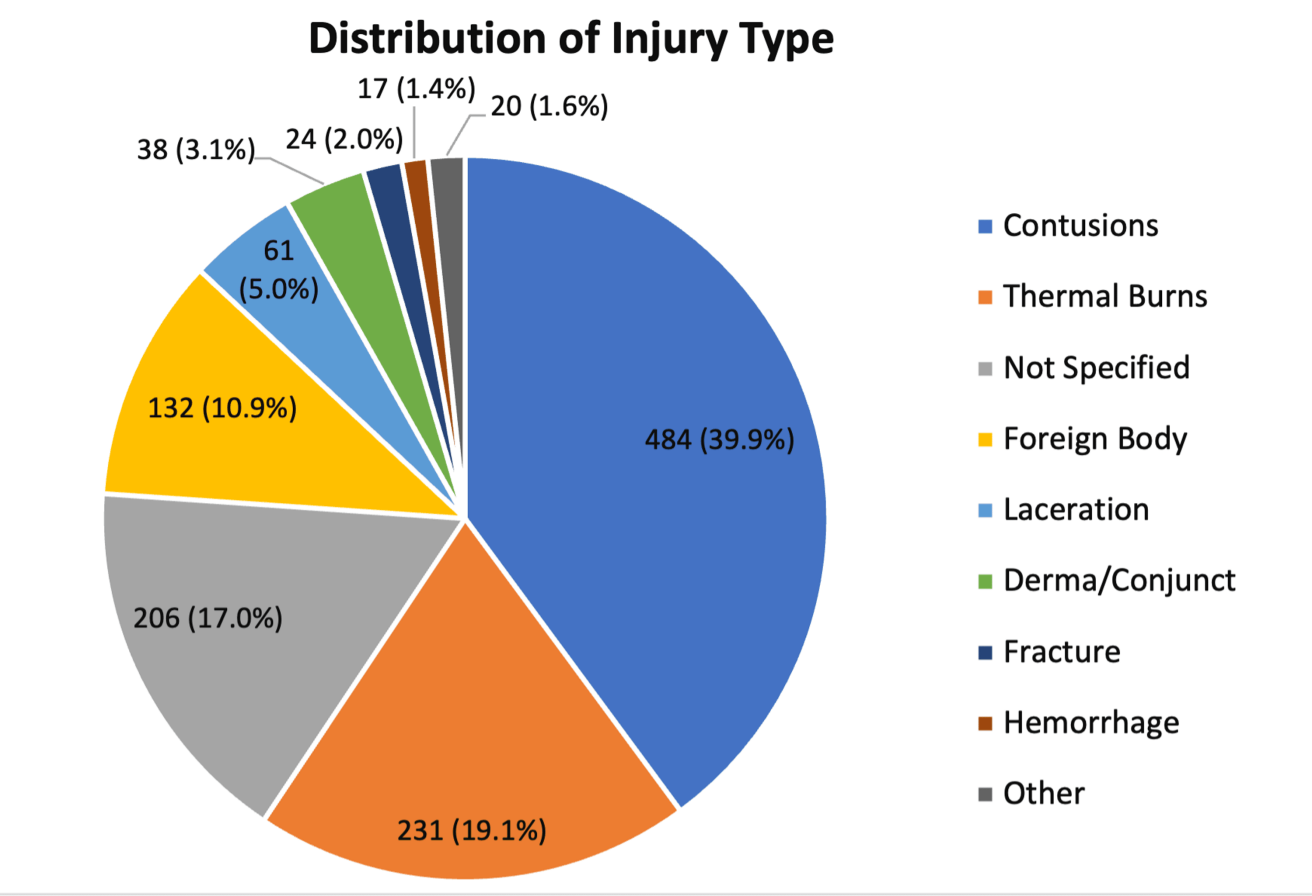
Pie chart illustrating the prevalence of each reported injury type. “Not Specified” injuries did not have a description of the injury type, whereas “Other” injuries are a collection of a small sample of injuries, including chemical burns, non-specified burns, puncture wounds, radiation injuries, amputation, hematoma, and internal injury.

Lastly, the data were stratified based on the reported injury type. Contusions were the most common injury type, accounting for 484 (39.9%) cases. Thermal burns were the second most prevalent injury type (231, 19.1%), followed by injuries without a documented specific diagnosis (206, 17.0%). The “Other” category included 20 (1.6%) injuries with low individual frequencies, comprising chemical burns (n = 9), unspecified burns (n = 3), puncture wounds (n = 3), radiation injuries (n = 2), amputation (n = 1), hematoma (n = 1), and internal injury (n = 1) (Figure 5).

## Discussion

This 20-year national analysis identified an estimated 41,708 emergency department visits for firework-related ocular injuries, with marked seasonal clustering in July and January around Independence Day and New Year’s Day. These findings are consistent with a prior national analysis that reported approximately 34,000 ocular firework injuries from 1999 to 2017 using NEISS data [3].

From 2002 to 2021, the overall number of injuries remained relatively stable, showing a statistically insignificant negative correlation (p = 0.268). Interestingly, this stability contrasts with reports from the American Pyrotechnics Association (APA) that show an increase in firework sales during the same timeframe (Publicly Available Report: American Pyrotechnics Association. U.S. Fireworks Industry Revenue Figures Breakdown by Industry Segment 2000-2021; accessed February 12, 2023). While causation cannot be inferred, this divergence may suggest improvements in safety practices or product regulation. Supporting this, injuries per 1,000 pounds of fireworks decreased from 4.6 in 2002 to 2.7 in 2021, even as firework consumption increased by 126% (Publicly Available Report: American Pyrotechnics Association. Fireworks Related Injury Rates, 1976-2021; accessed February 12, 2023). While this data reflects all injury types, not exclusively ocular injuries, a previous analysis of eye-specific injuries from 1999 to 2017 reported no statistically significant change in the annual rate of firework-related eye injuries [3].

Despite overall stability, there were moderate spikes in injuries in 2005, 2020, and 2021. APA data indicate that consumer firework sales increased by 19.2% from 2004 to 2005, 48.2% from 2019 to 2020, and 5.7% from 2020 to 2021, with the majority purchased for at-home use (Publicly Available Report: American Pyrotechnics Association, 2023). Consumer fireworks, as opposed to professional display fireworks, are more likely to be handled by untrained individuals.

The COVID-19 pandemic likely contributed to the spikes in 2020 and 2021. Widespread cancellations of public firework displays led many individuals to purchase consumer fireworks for private shows. This is corroborated by a Californian environmental study that documented elevated PM2.5 levels, a proxy for firework activity, on the Fourth of July in 2020 despite public show cancellations [4]. These findings suggest that increased consumer firework use and at-home displays during social restrictions may have contributed to higher injury rates during these years. However, this explanation does not account for the moderate increase observed in 2005, which may reflect other unmeasured factors.

A more detailed analysis of injury type provides additional insight into firework-related ocular trauma. Contusions, which include iris sphincter ruptures, uveitis, lens luxation, elevated intraocular pressure, hemorrhages, choroid ruptures, retinal tears, and detachments, accounted for the majority of injuries (484, 39.9%) (Figure 5). However, previous investigators have reported that thermal burns represent the most common firework-related ocular injury, accounting for 633 of 1,007 cases (62.9%) [3]. This study differs methodologically from previous work by incorporating both globe (code 77) and facial (code 76) injury codes with narrative confirmation of ocular involvement. This broader capture likely increased documentation of peri-ocular injuries that might otherwise be excluded from eye-specific codes, potentially explaining the higher proportion of periocular contusions.

Thermal burns and non-specified injuries were the second and third most prevalent injury types. This distribution aligns with prior studies reporting ocular burns, eyelid injuries, corneal trauma, and vitreous hemorrhage as common outcomes [1,3].

Children were disproportionately affected, comprising 756 (62.3%) cases. This finding is consistent with prior national analyses, including Shiuey et al., who reported that 664 (65.9%) ocular injuries occurred in individuals under 18 years of age [3]. In our cohort, the median age at injury was 14 years for males and 15 years for females, underscoring the early age at which these injuries occur.

Early-onset ocular trauma carries significant public health implications, as visual impairment during childhood may adversely affect educational attainment, psychosocial development, employment opportunities, and overall quality of life [5-8]. Children with visual impairment often experience diminished functional ability and reduced participation in social and academic activities [6,8].

The persistence of this pediatric predominance across multiple decades suggests that existing public health messaging and regulatory strategies may be insufficient to protect minors. Given the continued growth in consumer firework sales and the predominance of injuries associated with home use, targeted preventive efforts should focus on promoting safe handling practices, restricting adolescent access to consumer fireworks, and encouraging attendance at professionally supervised displays rather than private use [1,3,9].

Increased utilization of protective eyewear represents a potentially modifiable prevention strategy. Although not currently mandated in the United States, safety goggles could significantly reduce ocular trauma from projectiles, thermal burns, and chemical exposure. Prior literature emphasizes the limited use of protective eyewear in firework-related eye injuries, highlighting their preventable nature [10,11]. Integrating safety equipment with firework sales and public education campaigns could mitigate these risks for both users and bystanders.

This study has several important strengths. By incorporating both globe (code 77) and facial (code 76) injury codes with manual narrative confirmation, we identified 17.3% of ocular injuries that would not have been captured using eye-specific codes alone, highlighting the importance of comprehensive case ascertainment in NEISS-based analyses. Additionally, the use of a nationally representative database spanning 20 years strengthens the generalizability of these findings across diverse geographic regions and demographic groups. However, several limitations should be acknowledged. NEISS captures only injuries presenting to emergency departments, potentially underestimating the true burden of firework-related ocular trauma. The database also lacks detailed clinical information regarding injury severity, treatment course, surgical intervention, and long-term visual outcomes. Furthermore, although manual narrative review improved case identification, this process introduces potential subjectivity, and inter-reviewer reliability was not formally assessed. As with all registry-based studies, findings remain dependent on the accuracy and completeness of emergency department coding and documentation.

## Conclusions

In conclusion, this 20-year analysis of the NEISS database demonstrates that firework-related eye injuries remain a persistent public health concern in the United States, accounting for an estimated 41,708 emergency department visits from 2002 to 2021. Although the annual incidence of reported injuries remained relatively stable despite substantial increases in fireworks consumption, distinct temporal and demographic patterns were observed. Injuries occurred predominantly in July and disproportionately affected males and individuals 18 years of age and younger, with children and adolescents representing the majority of cases. Contusions were the most commonly reported injury type, particularly when periocular injuries coded under facial classifications were included, underscoring the importance of comprehensive injury capture when evaluating ocular trauma trends. These findings highlight the continued vulnerability of pediatric populations and the need for targeted prevention strategies. Increased consumer firework use, particularly during periods of reduced public displays such as the COVID-19 pandemic, may contribute to episodic rises in injury incidence. Public health initiatives focused on education, stricter regulation of consumer fireworks, and the promotion or provision of protective eyewear could meaningfully reduce preventable ocular trauma. Future research examining state-level legislation and differentiating injuries by firework type may further clarify risk factors and inform evidence-based policy aimed at preserving vision and reducing long-term morbidity associated with firework-related eye injuries.
